# 563. Safety and Immunogenicity of mRNA-1273.815 in COVID-19 Vaccine-Naïve Children 2 Through 4 Years of Age: Results From a Phase 3, Open-Label Trial

**DOI:** 10.1093/ofid/ofaf695.172

**Published:** 2026-01-11

**Authors:** Avika Dixit, Roozbeh Sharif, Rosane Poston, Greer Chambers, Anne Yeakey, Heather Williams, Bethany Girard, Wen Zhou, Wenqin Xu, Spyros Chalkias, Frances Priddy, Rituparna Das

**Affiliations:** Moderna, Inc., Cambridge, Massachusetts; REX Clinical Trials, LLC Beaumont, Beaumont, Texas; Moderna, Inc., Cambridge, Massachusetts; Moderna, Inc., Cambridge, Massachusetts; Moderna, Inc., Cambridge, Massachusetts; Moderna, Inc., Cambridge, Massachusetts; Moderna, Inc., Cambridge, Massachusetts; Moderna, Inc., Cambridge, Massachusetts; Moderna, Inc., Cambridge, Massachusetts; Moderna, Inc., Cambridge, Massachusetts; Moderna, Inc., Cambridge, Massachusetts; Moderna, Inc., Cambridge, Massachusetts

## Abstract

**Background:**

Due to infection and/or immunization, SARS-CoV-2 seroprevalence rates in children aged 2-4 years have increased and are now comparable to those aged ≥5 years; these children may benefit from a single dose vaccine regimen currently recommended for those aged ≥5 years. Here, we present results from a phase 3 study evaluating the effectiveness of a single dose of an XBB.1.5-containing vaccine (mRNA-1273.815) in children aged 2-4 years.

**Figure:**
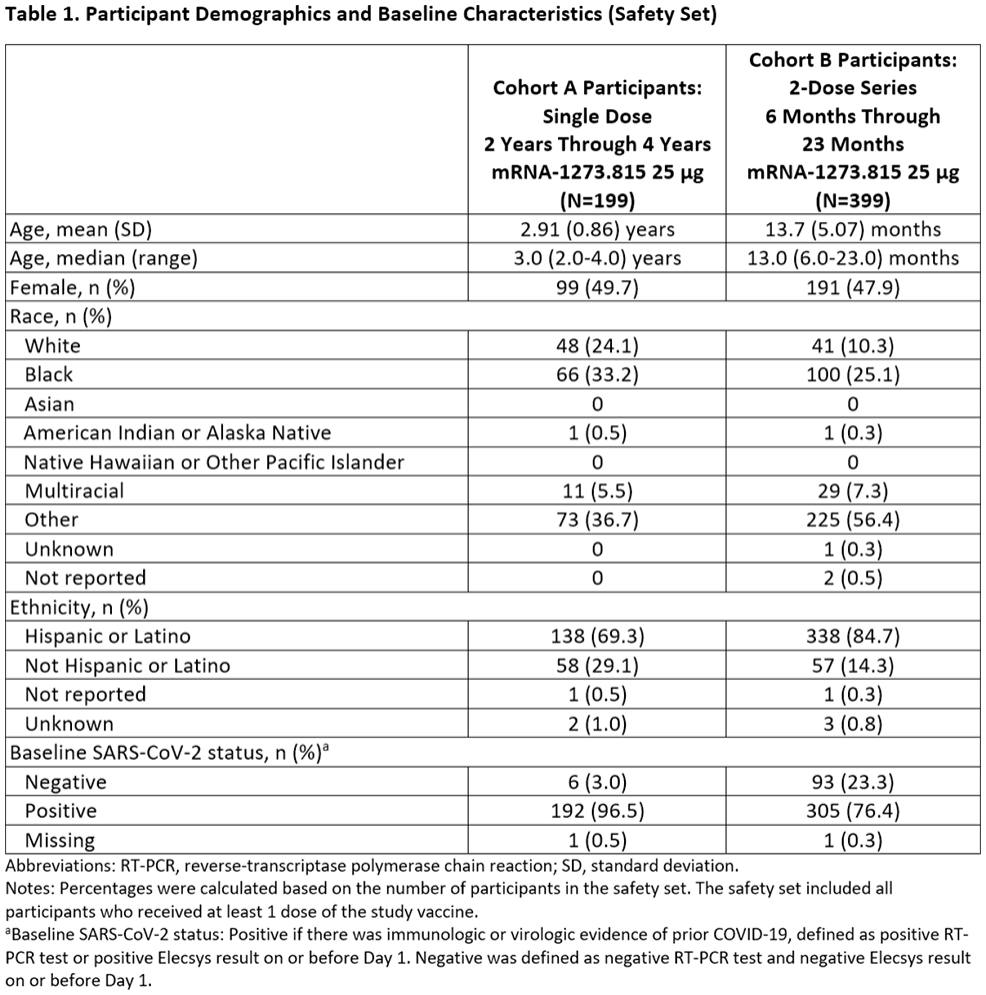

**Figure:**
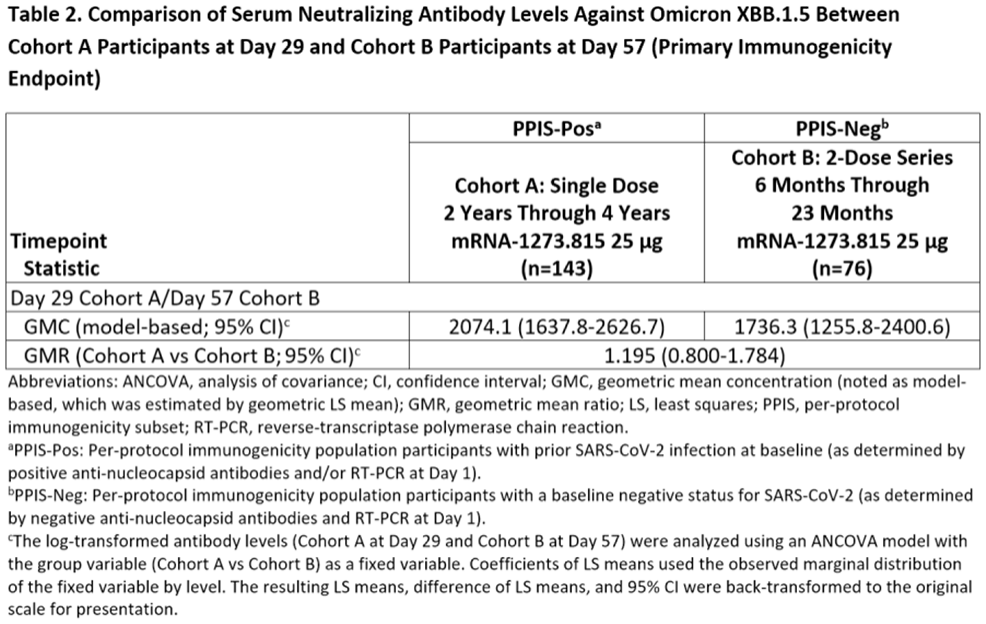

**Methods:**

An open-label trial (NCT05436834) evaluated safety and immunogenicity of monovalent mRNA-1273.815 in COVID-19 vaccine-naïve participants aged 2-4 years (Cohort A; single 25 µg dose). Effectiveness was inferred based on comparison of immune responses (measured by geometric mean concentrations of neutralizing antibodies against XBB.1. 5) in baseline SARS-CoV-2 positive (Pos) Cohort A participants (Per-protocol immunogenicity subset-Pos [PPIS-Pos]) to vaccine-naïve baseline SARS-CoV-2 negative (Neg) participants aged 6-23 months (Cohort B PPIS-Neg; 25 µg 2-dose series). Noninferiority (NI) of the single dose regimen was considered established if lower bound of the 95% confidence interval (CI) of the geometric mean ratio (GMR: Day 29 Cohort A vs Day 57 Cohort B) was >0.667. Safety was assessed through 3 months after vaccination.

**Results:**

A total of 199 participants (Cohort A) and 399 participants (Cohort B) received study vaccine; median ages were 3 years and 13 months, respectively (Table 1). Safety profile of mRNA-1273.815 in both cohorts was similar to the established safety profile of mRNA-1273 vaccines in the respective age groups and generally well-tolerated. In the primary immunogenicity analysis against XBB.1.5, the GMR of a single 25 µg dose of mRNA-1273.815 (Cohort A PPIS-Pos) compared to a 25 µg 2-dose series of mRNA-1273.815 (Cohort B PPIS-Neg) was 1.195 (95% CI, 0.800-1.784; Table 2), meeting the prespecified NI criterion.

**Conclusion:**

No new safety concerns were identified in children aged 2-4 years and 6-23 months who received mRNA-1273.815. Success criteria for the primary immunogenicity endpoint were met, establishing the effectiveness of a single 25 µg dose. These data support lowering the age recommendation for a single dose of mRNA-1273 COVID-19 vaccines from ≥5 years to ≥2 years, regardless of prior immunization status.

**Disclosures:**

All Authors: No reported disclosures

